# Herpes zoster in outpatient departments of healthcare centers in India: a review of literature

**DOI:** 10.1080/21645515.2021.1968737

**Published:** 2021-09-14

**Authors:** Anil Patki, Agam Vora, Raunak Parikh, Shafi Kolhapure, Ashish Agrawal, Resham Dash

**Affiliations:** a Deenanath Mangeshkar Hospital, Dermatology, Pune, India; b Dr. R.N. Cooper Municipal General Hospital, Chest and Tuberculosis, Mumbai, India; c Medical Affairs Department, GSK, Wavre, Belgium; d Medical Affairs Department, GSK, Mumbai, India; e Medical Affairs Department, GSK, Hyderabad, India; f Medical Affairs Department, GSK, New Delhi, India

**Keywords:** Herpes zoster, older adults, post-herpetic neuralgia, India, vaccination

## Abstract

In India, although incidence of Herpes zoster has not been assessed, regional cases have been reported. We revisited the peer-reviewed literature on clinical cases of HZ to depict the trends in population characteristics, disease presentation, and predisposing factors for the disease in India. Systematically conducted literature search yielded 27 studies, published between January 2011 and May 2020, reporting 3124 HZ clinical cases, with high proportions in older adults (>50 years of age: 15.0–81.3%). Thoracic dermatome was consistently reported as the most frequent site affected by HZ (38.9–71.0%). Post-herpetic neuralgia and secondary bacterial infections were the two most frequent complications (10.2–54.7% and 3.5–21.0%, respectively). Despite the paucity of data and gaps in the reporting of HZ cases, available evidence indicate that the disease causes an important burden to older adults in India, suggesting that preventive strategies, along with recommendations to healthcare practitioners, can help mitigate the burden of HZ.

## PLAIN LANGUAGE SUMMARY

What is the context?

- Herpes zoster is caused by reactivation of the varicella virus in sensory gangila usually during older age.
- The most common complication involve skin and neurological disorders, such as post herpetic neuralgia.
- Herpes zoster can impact quality of life for months or even years (especially for those >50 years of age).
- In India, there is a lack of population-based studies on herpes zoster to reflect burden of disease.

What is new?

- We reviewed the literature on clinical cases of herpes zoster in India from last decade and found that:
- High propotions of older adults (>50 years of age) are reported to have the disease.
- Thoracic dermatomes and post herpetic neuralgia are common clinical presentation and complication.
- Post herpetic neuralgia is observed more frequently in older patients.

What is the impact?

- Our review highlights sigificant number of cases of herpes zoster are reported and the disease causes a substantial burden to older adults in India.
- In view of the growing elderly population in India, the finding of greater proportion of cases in >50 years of age holds importance.
- Implementation of preventive strategies along with guidance to healthcare pracitioners can help prevent the disease and complications in vulnerable population.

## Introduction

Herpes zoster (HZ) disease, also known as ‘Shingles,’ usually presents as a painful, vesicular dermatomal rash.^1^ It results from the reactivation of the latent varicella-zoster virus in sensory ganglia.^2^ The factors that trigger reactivation of dormant virus are not fully elucidated, though decline in cell-mediated immunity with age or immunosuppressive conditions or treatments play an important role.

HZ episodes may lead to complications and sequelae, including, neurological disorders like post-herpetic neuralgia (PHN), limb paralysis, cerebrovascular disorders like stroke, cardiovascular disease such as myocarditis, and serious skin alterations such as intense scarring.^1,3,4^ The most common complication encountered is PHN, when the pain accompanying the rash persists for months or even years after the initial episode. The pain can be debilitating and lead to physical disability, emotional distress, and sleep disorder.^5^ Worldwide, estimates for the risk of PHN following HZ episode range between 5% and 30%.^6^ Herpes zoster ophthalmicus (HZO) is another presentation of the disease that can lead to ocular complications, often causing decreased visual acuity or even blindness.^3,7^

Treatment of acute HZ is primarily designed to manage pain, and hasten recovery.^3^ Pain is usually managed with analgesics but may require strong opioids.^8^ Antiviral agents such as acyclovir, famciclovir, and valaciclovir are commonly used in the treatment of the disease.^3,8^ Local management of skin lesions may help in alleviating the discomfort and preventing the development of long-lasting skin lesions. For patients suffering from PHN, treatment comprises anticonvulsants, tricyclic antidepressants, topical therapies, and opioids.^4,9^

Many studies have been conducted across different parts of the world to study the incidence rates of HZ.^4,6,10^ In Asia, the overall HZ incidence has been estimated at 5.0 per 1000 person-years (PY).^4^ Stratification by age groups yielded median values of 2.0–3.1, 4.3–5.2, and 7.4–13.8 per 1000 PY in the 0–20, 20–50 and in >50 years old populations, respectively.^4^ To the best of our knowledge, there are no population-based studies with potential to provide incidence data specific to India. While there is study-to-study variation due to differences in methodology, location appears to have little impact on the incidence of HZ, which is similar in different regions of the world.^6,11–13^

The aim of this work was to better understand the clinical profile of HZ disease reported in India. To achieve this, literature was collected through searches carried out systematically in the PubMed and Embase databases for the period of Jan 2011 to May 2020. An additional search was performed in Google Scholar which is described in the literature search section. We have described the trends in demographic characteristics of HZ patients, clinical presentation of the disease, complications and predisposing factors reported in Indian studies published between 2011 and 2020.

## Methods

Research articles in English language published since 2011 were searched for “zoster” and “India” in all fields on May 20^th^ and 26^th^, 2020 in PubMed and Embase, respectively. An additional search was performed in Google Scholar for “study” and “herpes zoster” (all in title, including citations) on January 8^th^, 2021 to identify additional literature not indexed in PubMed nor Embase. Screening of titles, abstracts, and full texts was performed by two independent reviewers. Any discordance about the final selection of articles was collegially resolved according to the pre-specified inclusion/exclusion criteria. Data were collated for descriptive purpose.

### Inclusion criteria

Articles reporting analytical, prospective observational, cross sectional, retrospective reviews, and time bound studies conducted in either dermatology, medicine and/or ophthalmology departments of various medical colleges and tertiary care centers across India were included. Only studies reporting data from multiple HZ patients, providing quantitative data about demographics, various disease presentations, complications or risk factors in India were selected.

### Exclusion criteria

Individual case reports, case series not providing quantitative analysis, scholar theses, editorials and letters to editors were excluded from the review. Articles reporting interim analyses of studies for which the final analysis was already included were excluded from the review to avoid inclusion of redundant datasets. Articles published before 2011 and after May 2020 were excluded from the Google scholar search to match the settings of the Embase and PubMed searches.

### Literature search

There were 242 and 528 records retrieved from PubMed and Embase respectively (Supplementary Figure 1). After elimination of 163 duplicate records, titles and abstracts of 607 unique records were screened. A total of 14 articles were included from this initial search.^14–27^ A search for additional literature in Google Scholar, was carried out due to limited number of articles retrieved in the initial search. Post Google scholar search, 13 additional articles were further added.^28–40^ Also the 2013 paper from Nithyanandam et al.^25^ was a post-hoc analysis, so we retrieved the data from the primary publication.^41^ Data were extracted from the 27 relevant articles for review.

## Results

### Characteristics of included studies

**Figure 1.**
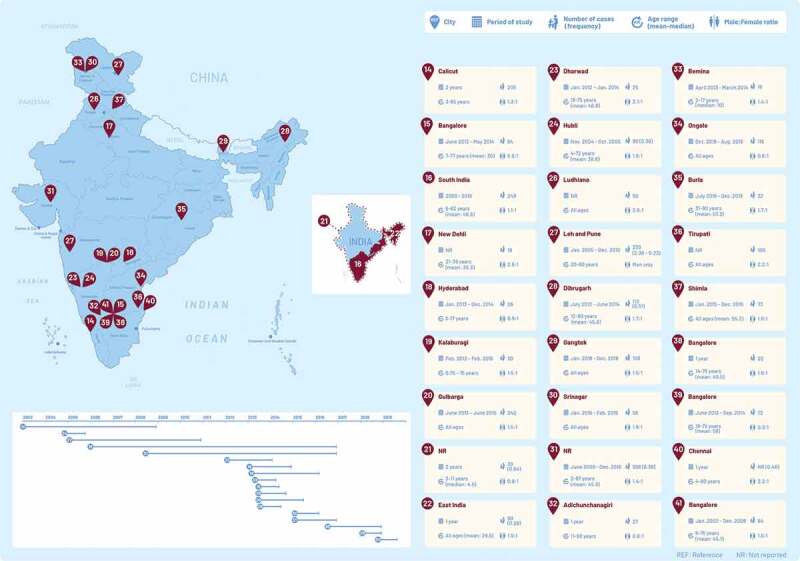
Study location, number of cases (including frequency among outpatients department visits) and their characteristics (age descriptors and sex ratio) from the retrieved peer-reviewed literature on herpes zoster in India (January 2011 – May 2020). Interactive version of the figure available on Figshare Supplemental data for this article can be accessed on the publisher’s website at *[https://doi.org/10.6084/m9.figshare.15156114.v1].*

A total of 27 studies collectively reporting 3124 HZ patients (range 18 to 938) conducted between 2003 and 2019 were retrieved. Location and period of these studies along with demographic characteristics of the HZ patients are reported in Figure 1. Key outputs extracted from all studies are reported in Supplementary Table 1.

NR: non reported This map is not to scale and is for generalized illustration only and is not intended to be used for reference purposes. The representation of political boundaries and the names of geographical features do not necessarily reflect the position of GSK on international issues of recognition, sovereignty, jurisdiction or nomenclature. While due care and caution has been taken to ensure that the map projected is free from mistakes or omissions, GSK, its directors and officers will not be liable for any damages whatsoever.

Of the retrieved studies, 24 were prospective cross-sectional and three were retrospective (Supplementary Table 1). Nine studies either did not mention or failed to report the study period. Clinical diagnosis and Tzanck smear tests were mostly used as case definition for HZ, though this information was not systematically reported. Most studies (n = 21) included patients of all ages, while four studies reported HZ cases in the pediatric population only.^18,19,21,33^ Eight studies reported exclusively on HZO cases.^16,17,23,32,35,37,38,41^

Of the 27 publications, seven^21,22,24,27,28,31,40^ provided proportions of HZ cases among all patients who attended outpatient visits in the considered departments, ranging between 0.28 and 2.36% (Figure 1). For example in the study with the largest sample size conducted in a rural hospital in Gujarat, 938 HZ cases were reported between June 2008 and December 2016 among all dermatology outpatients, yielding a proportion of 0.38%.^31^

### Demographics

#### Age range

**Figure 2.**
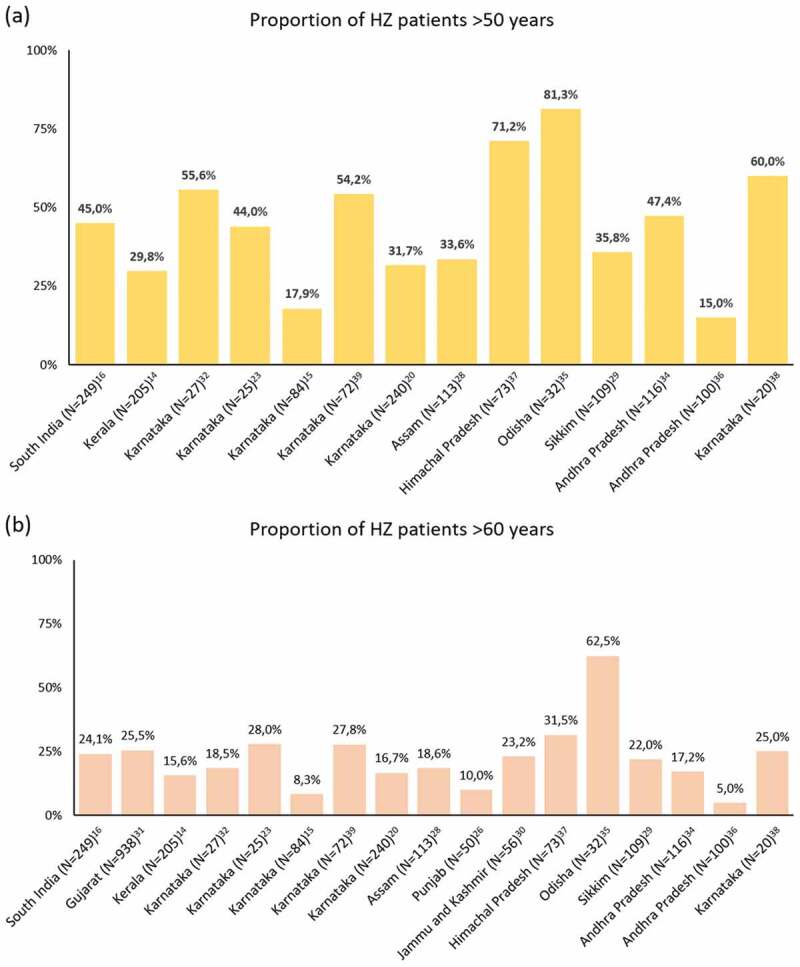
Proportions of HZ patients older than 50 years (a) and 60 years (b) of age in the retrieved peer-reviewed literature.

HZ: herpes zoster; N: total number of patients in the study.

As seen in Supplementary Table 1, eleven studies that included patients of all ages reported their mean age ranging between 29.6 and 57.3 years.^15,16,22–24,28,31,35,37,38,41^ Roughly half of the studies reported proportions of patients by age groups. Proportions of patients with HZ in >50 and >60 years ranged from 15.0%^36^ to 81.3%^35^ and from 5.0%^36^ to 62.5%,^35^ respectively. The very low proportions observed in the studies conducted by Usha et al.^36^ and Aggarwal et al.^15^ is at odd with other studies. It is noteworthy that the latter was conducted in a military hospital, explaining the high proportion of young adult male patients.^15^ This selection bias is however not discussed by the authors.^15^ Little details are provided by Usha et al. regarding the selection of the 100 patients included in the study, presenting a high risk of bias for the presented population.^36^ The distribution across studies for both age groups is depicted in Figure 2. The largest proportions were reported in a study conducted on 32 HZO patients in Odisha during 2016–2018, where 81.3% and 62.5% of the patients were older than 50 years and 60 years of age, respectively.^35^ In the retrieved study having the largest number of patients (N = 938), conducted between 2008 and 2016 in rural Gujarat, 25.5% of the cases occurred in individuals older than 60 years.^31^

#### Sex ratio

Male:female ratio ranged between 1.1:1 and 1.9:1 in 15 studies (Figure 1).^14,16,19,20,22,24,28–31,33,35,37,38,41^ Six studies found the ratios to be in the order of 2:1,^15,17,23,26,36,40^ Five studies, however, reported more women patients with HZ than men, in the range of 0.6:1 to 0.9:1.^18,21,32,34,39^

#### History of varicella infection and vaccination

Eleven studies reported the proportion of HZ patients with a known history of varicella infection (chickenpox).^14,17–22,24,28,31,33^ Proportion of patients who had chickenpox ranged between 22.7%^31^ and 79.6% (Supplementary Table 1).^28^ Varicella vaccination history was documented in only four studies,^18,19,21,33^ and proportions were in the range of 0.0%^33^ to 30.8%^18,21^ of cases.

#### Predisposing factors for HZ disease

Besides advanced age, human immunodeficiency virus (HIV) seropositivity, diabetes mellitus, prolonged steroid use, and malignancies with therapy were the frequently associated predisposing/co-morbid conditions in the retrieved studies (Supplementary Table 1). HIV status of the enrolled population was clearly stated in 23 studies.^14,15,17–24,26–28,31,32,34–41^ For other studies, HIV status was either not assessed or mentioned. The reported frequency of HIV seropositivity among the studied patients varied between 0%^22,27,38^ and 44.4%.^17^ Diabetes mellitus was reported in 15 studies^14,16,20,22,23,27–29,32,34–39^ with a percentage range of 0%^27^ to 56.3%.^35^ The highest proportion of diabetes (56.3%) was reported in a study conducted in the Ophthalmology department of a tertiary hospital in Odisha where 20/32 patients (62.5%) were older than 60 years of age.^35^

Immunocompetence level is not assessed or clearly reported in all studies. Moreover, some authors considered diabetes as an immunocompromising condition while others did not. According to authors’ assessments, proportion of immunocompromised patients varied between 0%^27^ and 44.4%.^17^ However, given the high uncertainty of the data and possible selection bias, data on immunocompromised population should be interpreted with caution.

### Disease presentation

#### Prodromal symptoms

Twenty studies described the prodromal symptoms experienced by the patients (Supplementary Table 1).^14,16,18–24,26,28,31,32,34–39,41^ Reported prodromal symptoms included segmental pain (58.9%–100%),^24,39^ fever (2.5%–44.4%),^22,31^ headache (3.4%–10.0%),^20,31^ itching (8.0%–45.5%),^22,28^ burning sensation (13.3%–68.9%),^19,34^ and paresthesia (6.2%–50.0%).^26,28^ In studies focused on HZO patients,^16,17,23,32,35,37,38,41^ the reported prodromal symptoms were eye watering (33.3%–58.9%),^32,37^ lid swelling (18.5%–87.5%),^32,35^ and diminution of vision (25.0%–37.0%).^32,38^

#### Rash characteristics

Eighteen studies reported dermatomal distribution as per site/location in HZ patients (Table 1). Thoracic dermatome was consistently reported as the most frequent dermatome involved (38.9%–71.0%).^27,39^ Other commonly affected sites were the cranial (3.3%–28.3%),^14,19,28^ cervical (4.0%–23.8%),^27,36^ and lumbar (5.5%–35.0%) regions.^29,33^ Three studies reported cases of multi-dermatomal involvement.^29,31,36^ Sharma and Sharma observed multi-dermatomal presentation in 8.25% of the 109 reported cases.^29^ Among the 100 patients reported by Usha et al., 42% had multi-dermatomal involvement, among which 87.5% were living with HIV.^36^ Vora et al. noticed that among 938 HZ patients, 43 cases (4.6%) had multiple dermatomes involved.^31^

**Table 1.** Proportions of cervical, cranial, lumbar, and thoracic dermatomes among HZ patients across the retrieved studies

Authors	N	Thoracic	Cranial	Cervical	Lumbar
Abdul Latheef and Pavithran, 2011^14^	205	42.4%	28.3%	12.2%	
Adhicari and Agarwal, 2017^28^	113	45.1%	28.3%		15.0%
Aggarwal and Radhakrishnan, 2016^15^	84	65.5%	10.7%	11.9%	
Malkud et al., 2016^20^	240	44.2%	24.6%	12.5%	9.2%
Mondal et al., 2019^22^	90	46.7%			
Naveen et al., 2011^24^	90	46.7%	18.9%	13.3%	14.4%
Puri, 2016^26^	50	40.0%	24.0%	8.0%	16.0%
Sharma and Sharma, 2019^29^	109	40.4%	22.9%	23.8%	5.5%
Singh et al., 2018*^27^	173 vs 66	60.0% vs 71.0%	20.0% vs 9.0%	4.0% vs 5.0%	11.0% vs 14.0%
Mitra et al., 2018^21^	39	51.3%			
Vora et al., 2018^31^	938	39.9%	25.1%	18.0%	12.1%
Katakam et al., 2016^18^	26	53.8%	15.4%		
Malkud and Dyavannanavar, 2017^19^	30	56.7%	3.3%	16.7%	20.0%
Lanker et al., 2015^33^	19	55.0%	5.0%		35.0%
Naik, 2019^34^	116	53.4%	17.2%	19.0%	6.0%
Usha et al., 2015^36^	100	51.0%	12.0%	21.0%	
Rachana et al., 2017^39^	72	38.9%	22.2%	5.6%	30.6%
Sundaram et al., 2012^40^	NR	55.0%	21.5%	4.6%	18.4%

*High altitude population versus (vs) plain population. N: total number of patients; NR: not reported.

#### Pain characteristics

Pain was a common symptom in many studies.^14,18–22,24,26,28,30,31,34,37–39,41^ Pain preceded vesicles onset in 60.4% of the patients recruited in Karnataka by Malkud et al.^20^ In the same study, 15 out of 240 patients (6.3%) experienced intermittent radicular pain and 10 patients (4.2%) suffered continuous pain.^20^

In the study by Vora et al., burning pain affected 720/938 HZ patients (76.8%), which was by far the most common type of pain encountered; other types of pain were itching pain (8.0%), pricking pain (7.4%) and throbbing pain (5.2%).^31^ Burning pain (18.3%) and pricking pain (54.2%) were also described in the study by Rachana et al. on 72 HZ patients.^39^ Burning pain was also denoted as a common finding in the three studies reported by Adhicari and Agarwal, Gupta and Sareen, and Mitra et al.^21,28,37^

### Complications

Among the retrieved studies, 21 publications provided data on associated complications (Supplementary Table 1).^14–18,20–24,26,28,30,32–38,41^ PHN (10.2%-54.7%)^14,41^ and secondary bacterial infections (3.5%–21.0%)^28,36^ were the most frequently reported complications. Other complications included scarring (including keloids),^14,20,24,28,34,36^ paresthesia with motor involvement,^14,15,20,30^ sensory and hearing loss,^16,32,34,35,37,41^ Ramsay Hunt syndrome,^26,30^ and pigmentary changes.^14,20,22,28,36^ Among HZO patients, corneal lesions were common.^16,17,23,41^ HZO patients also suffered a wide range of complications including visual impairment,^23,32,35,37,41^ and secondary glaucoma (detailed in Supplementary Table 1).^17,32,35,37,38^

#### Post-herpetic neuralgia

PHN accounted for 10.2% to 54.7% of the reported complication among HZ patients (Supplementary Table 1),^14,41^ and its incidence was found to be higher among the older individuals (>50 years old) in the study of Puri.^26^ Also, as per Adhicari et al., maximum cases of PHN were concentrated in the age group 41–70 years old.^28^ Similarly, Malkud et al.^20^ reported that overall proportion of PHN among the 240 HZ patients was 10.4%, and that half of HZ patients 60 years or older developed this complication.^20^

PHN was also observed with higher frequency in immunocompromised patients. In a study conducted by Gupta et al.,^17^ proportion of PHN was higher among patients living with HIV (n = 6/8: 75%) when compared to the rest of HZ patients (n = 1/10: 10.0%). Usha et al. also reported a greater proportion of PHN cases in HIV seropositive patients (n = 8/32: 25%) relative to HIV seronegative patients (n = 7/68: 12.9%).^36^

### Herpes zoster ophthalmicus

HZO, the form of HZ affecting the first branch of the trigeminal nerve, i.e. the ophthalmic nerve, may include additional ocular symptoms and complications. Eight studies specifically described HZO and its associated complications in detail (Supplementary Table 1).^16,17,23,32,35,37,38,41^ Acute corneal epithelial lesions (64.1%), reduced corneal sensation (67.2%) and uveitis (48.4%) were some of the serious complications reported in a study of 64 patients.^41^ In another study (n = 25 HZO patients), 76.0% suffered lid edema while 52% had decreased corneal sensitivity.^23^ Also, impaired visual acuity was reported in 40% of the patients in the same study.^23^ Lid (52.6%) and corneal (56.6%) involvement, along with PHN (30.9%) were also reported in a retrospective study (n = 249 HZO cases) between 2006 and 2016 in two tertiary referral eye centers.^16^ Anterior uveitis was the most frequent presenting symptom (>50% of cases) seen.^16^ In a separate study of 18 HZO, 83.3% of the patients manifested corneal involvement.^17^ In the same study it was mentioned that visual acuity was better in patients with HIV-negative status than in those living with HIV.

## Discussion

This is the first large scale review of the published literature to ascertain the occurrence of HZ and its complications in India, as well as gaps in available data about the disease in India. Acknowledging the fact that HZ is not notifiable and in the absence of population-based epidemiological studies, along with poor surveillance system, it is important to fall back on a literature review. Though studies in various outpatient settings of tertiary care centers differ in terms of design, methodology and other various aspects, the data are of significance as it gives a fair estimate of the course of disease, its characteristics, and complications. Nonetheless, possible recruitment bias and gaps in data regarding patients’ characteristics, disease presentation, as well as complication in the retrieved literature, impaired us from performing quantitative analyses and may be responsible for the differences in proportions of HZ in older adults, as well as rates of complications, PHN and bacterial infections.

This review notably indicates that the risk of HZ is significant in individuals older than 50 years of age, as observed in studies conducted in other parts of the world, and that HZ cases are seen by a variety of medical specialists (dermatology, ophthalmology, internal medicine, neurology, etc), depending on disease presentation. Proportions of HZ patients among patients with other conditions at dermatology outpatient departments, was between 0.28%^22^ and 2.36%.^27^ The proportions differ among studies mainly due to differences in characteristics of the population evaluated. Despite this fact, the pattern is similar to that reported in Asia-Pacific region (e.g. Australia: 1.81 HZ cases/1000 consultations, 2006–2012).^4^ This tends to indicate that proportion of HZ in India is similar to rates reported elsewhere.^4^ However, HZ proportions seen in this review are likely to be underestimated, mainly due to factors such as community and patient seeking behavior, physician practice, HZ severity, etc. It was moreover observed that HZ incidence rates are increasing in countries across Asia-Pacific (Korea 1994–2003: 3.0/1000 PY; Korea 2003–2007: 10.0/1000 PY; Taiwan 2000: 4.04/1000 PY; Taiwan 2009: 6.24/1000 PY),^4,42^ which is most likely attributable to rising incidence in aging populations.^4^ Similarly, a growing number of HZ cases is expected to occur in India in the coming years as the Indian population aged 50 years and above has quadrupled over the last years, and is expected to comprise 404 million people in 2036, representing 27% of the country’s projected population.^43,44^ Indeed, while HZ cases were reported across age groups, it was predominant in individuals >50 years of age (15.0–81.3%). This is in agreement with the literature where the age-related increased incidence of HZ is thought to result from the decline in cell-mediated immunity (immunosenescence).^13^ Besides advanced age, patients affected by chronic diseases are also fragile due to impairment of cell-mediated immunity.^45–47^ Accordingly, HIV, diabetes mellitus, malignancies, and other chronic conditions were the frequently reported ailments in patients with HZ. It is noteworthy that India had an epidemic peak of HIV around 2000 and that the prevalence trend is now declining, reaching 0.22% among adults aged 15 to 49 years in 2019.^48^

PHN is the most common complication of HZ. In present review, 10.2% to 54.7%^14,41^ of the patients accounted with PHN. Though the risk of PHN may vary by study design, age or definitions used for PHN, the proportion was in accordance with the reported numbers (5% to >30%), including studies from North and South America, Europe, the Middle East, as well as the Asia-Pacific region.^6,49^ The incidence of PHN was found to be higher among the older (>50 years old) HZ patients, in agreement with the reported increase in frequency and severity of PHN with advancing age.^50^ The pain and discomfort associated with PHN can be prolonged and disabling, diminishing the patient’s quality of life and ability to function.^4,51^

HZO is another relatively common presentation of HZ with a reported incidence of 10–15% of all herpes zoster cases.^6,52^ HZO and ocular complications were also reported in studies included in present review. As per peer-reviewed literature, wide range of eye complications, such as keratitis, uveitis and conjunctivitis have been reported in the range of 30% to 78%.^6^

In India, HZ is not a reportable disease and despite poor surveillance system, a good number of clinical cases were reported in this review. Also, in view of the growing elderly population in India, the finding of greater proportion of cases in >50 years of age holds importance. The evidence generated needs be further strengthened and one way would be to conduct nationwide population-based study that is critical to investigate the true burden and epidemiology of HZ disease in India. However, until that time, as HZ cases are seen by a variety of medical specialists and the disease can cause substantial morbidity among older adults, prevention strategies and recommendations or guidance from healthcare professionals (HCPs) can play a critical role.

**Figure 3.**
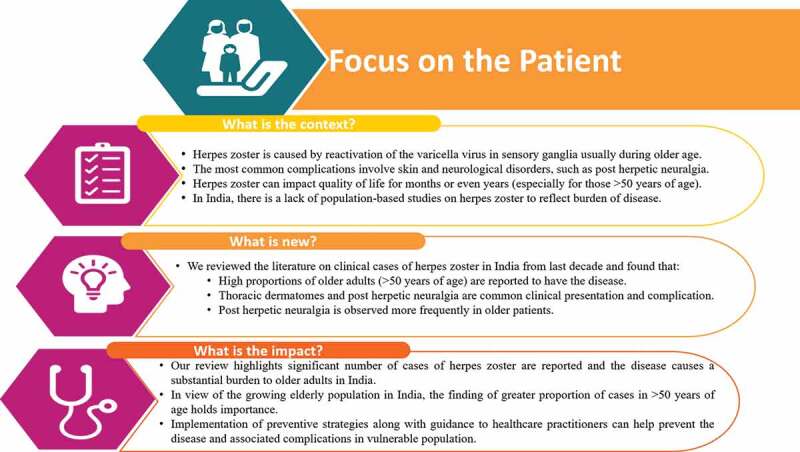
Plain language summary.

Figure 3 elaborates on the findings in a form that could be shared with patients by HCPs.

## Supplementary Material

Supplemental Material

Supplemental Material

## Figures and Tables

**Table:** 

HCP	healthcare professional
HIV	human immunodeficiency virus
HZ	herpes zoster
HZO	herpes zoster ophthalmicus
PHN	post-herpetic neuralgia
PY	person-year
