# Supplementary material for: Herpes zoster in outpatient departments of healthcare centers in India: a review of literature
Source: Hum Vaccin Immunother. 2021 Sep 14;17(11):4155–62. doi: 10.1080/21645515.2021.1968737 (PMC8828134; doi:10.1080/21645515.2021.1968737)
Supplement: Supplemental Material [file KHVI_A_1968737_SM7852.docx]

**Supplementary Table 1.** Design characteristics and main results of studies conducted in HZ and/or HZO patients in India, published between January 2011 and May 2020

| City, State (Author, year) | Setting | Study Design* | Study population | Comorbid conditions % (n)/ Immunocompromised (IC) cases % (n) | Prodromal symptoms/ Presenting symptoms, % (n) | Rash characteristics, % (n) | Pain characteristics, duration, intensity | PHN definition | Complications, % (n) |
| --- | --- | --- | --- | --- | --- | --- | --- | --- | --- |
| Bangalore, Karnataka (Nithyanandam et al. 2010)^1^ (reference **41** of main text) | Dermatology & Ophthalmology Dpt of Medical College Hospital | Prospective observational study, 6 months follow-up  Jan 2003–Dec 2008 | - HZO† pts - All ages - 64 pts - m:f 1.6:1 - Ages 6–75 yrs - Mean age (SD): 45.1 (17.9) yrs | HIV [IC]: 37.5% (24/64) | Rash (100%: inclusion criterion) | NR | Acute pain: 89.1% (57/64) | Any persistent  herpetic pain present 1 month after the  resolution of the rash | - Conjunctival involvement: 84.4% (54/64) - Reduced corneal sensation: 67.2% (43/64) - Corneal epithelial lesions: 64.1% (41/64) - PHN** 54.7% (35/64) - Uveitis: 48.4% (31/64) - Hutchinson sign 45.3% (29/64) - Visual loss: 34.3% mild, 4.7% moderate, 4.7% severe) - Corneal stromal lesions: 23.4% (15/64) |
| Hubli, Karnataka (Naveen et al., 2011)^2^ (reference **24** of main text) | Dermatology Dpt of Karnataka Institute of Medical Science | Prospective cross-sectional observational study  Nov 2004–Oct 2005 | - HZ† pts - All ages - 90 pts - m:f 1.6:1 - Ages 4–72 yrs - Mean age (SD): 38.8 (15.6) yrs - Chicken pox history: 53.3% (48/90) | - HIV [IC]: 37.8% (34/90) | - Pain: 58.9% (53/90) - Skin lesion: 16.7% (15/90) - Pre-herpetic neuralgia, mean (SD) duration: 2.1 (1.4) days | Dermatomal vesicular eruptions:^   - Thoracic: 46.7% (42/90) - Cranial: 18.9% (17/90) - Lumbar:14.4% (13/90) - Cervical: 13.3% (12/90) - Sacral: 6.7% (6/90) | NR | NR | - Scarring: 8.9% (8/90) - Recurrent zoster 5.6% (5/90) - Corneal ulcer: 2.2% (2/90) |
| Leh, Jammu and Kashmir – Pune, Maharashtra (Singh et al., 2018)^3^ (reference **27** of main text) | Dermatology Dpt of General Hospital (Leh) and Tertiary Hospital (Pune) | Retrospective cross-sectional observational study  Jan 2005–Dec 2010 | - HZ† immunocompetent pts - Adults - Only men - 173 pts (Leh) and 66 pts (Pune) - Ages: 20–60 yrs | [as per enrollment criteria immunocompromised, or pts with DM were excluded from enrollment  IC: 0%] | NR | \|  \| High altitude \| Plain area \| \| --- \| --- \| --- \| \| Thoracic \| 60.0% \| 71.0% \| \| Lumbar \| 11.0% \| 14.0% \| \| Cervical \| 4.0% \| 5.0% \| \| HZO \| 20.0% \| 9.0% \| | NR | NR | NR |
| South India (Babu et al., 2018)^4^ (reference **16** of main text) | Two Tertiary Eye Care Hospitals | Retrospective chart review  Patients records 2006–2016 | - HZO† pts - All ages - 249 pts - m:f 1.1:1 - Ages: 6–82 yrs - Mean age (SD): 46.8 (17.7) yrs - Aged ≥50yrs: 45.0% (112/249) - Aged >60yrs: 24.1% (60/249) | - DM: 13.3% (33/249) - HIV: (not reported but <5.6%) - IC: 5.6% (14/249) | Anterior uveitis: >50% | Active HZ lesions 80.3% (200/249), with right side of face common 49.4% (123/249) | NR | Pain for at least 3 months after onset of HZO | Pigmented keratic precipitates during activity: 63.4% (158/249)  Keratic precipitates: 57.4% (143/249)  Corneal involvement: 56.6% (141/249)  Absence of corneal sensation: 54.2% (135/249)  Presence of dry eye: 53.0% (132/249)  Lid involvement: 52.6% (131/249)  PHN††: 30.9% (77/249)  Granulomatous inflammation: 28.4% (40/249)  Increased intraocular pressure: 23.7% (59/249)  Abnormal iris pattern:15.3% (38/249)  Pupillary abnormalities: 14.9% (37/249)  Optic nerve abnormalities: 8.8% (22/249)  Scleral involvement: 8.0% (20/249)  Vitritis: 4.4% (11/249)  Abnormal extra ocular movements: 3.6% (9/249) |
| NR, (rural) Gujarat (Vora et al., 2018)^5^ (reference **31** of main text) | Dermatology Dpt of Tertiary Care Hospital | Cross-sectional observational study  June 2008–Dec 2016 | - HZ† pts - All ages - 938 pts - m:f 1.4:1 - Ages: 2–87 yrs - Mean age (SD): 45.9 (NR) - Aged >60 yrs: 25.5% (239/938) - Chicken pox history: 22.7% (213/938) | HIV [IC]: 4.3% | Overall in 93.9% cases:   - Itching: 43.6% (409/938) - Burning: 18.2% (171/938) - Tingling: 12.0% (113/938) - Paraesthesia: 11.0% (103/938) - Headache: 3.4% (32/938) - Watering eyes: 2.3% (22/938) - Fever: 2.5% (23/938) - Frontal sinusitis 0.9% (8/938) | Dermatomal vesicular eruptions:^   - Thoracic: 39.9% (374/938) - Trigeminal: 25.1% (235/938) - Cervical: 18.0% (169/938) - Lumbar: 12.1% (114/938) - Multiple dermatomal: 4.6% (43/938) | Overall 97.3% (913/938) with pain:   - Burning pain 76.8% (720/938) - Itching pain: 8.0% (75/938) - Pricking pain 7.4% (69/938) - Throbbing pain 5.2% (49/938) | NR | NR |
| Calicut, Kerala (Abdul Latheef and Pavithran, 2011)^6^ (reference **14** of main text) | Various Dpt of Medical College | Prospective observational study, 3 months follow-up  2 yrs | - HZ† patients - All ages - 205 pts - m:f 1.3:1 - Ages: 3–85 yrs - Aged >50 yrs: 29.8% - Aged >60 yrs: 15.6% (32/205) - Chicken pox history: 63.4% (130/205) | Overall comorbidities: 30.5% (62/205) cases :   - 9.8% (20/205) steroid therapy:asthma dermatitis, lepra, systemic lupus erythematosus, idiopathic thrombocytopenic purpura, erythema multiforme - 9.3% (19/205): Chemo-/radio-therapy for malignancies - 5.4% (11/205): DM - 4.9% (10/205): HIV - 1% (2/205): TB   IC: 29.3% (60/205) | Overall: 10.7 % (22/205) cases† | Dermatomal vesicular eruptions:^   - Thoracic: 42.4% (87/205) - Cranial: 28.3% (58/205) - Cervical: 12.2% (25/205) | Mild to severe segmental neuralgia: 94.6% | NR | Overall: 34.6 % (71/205) cases:   - SBI: 13.6% (28/205) - PHN: 10.2 % (21/205) - Scarring: 3.9% (8/205) - Depigmentation: 2.4% (5/205) - Severe ulceration: 2.0% (4/205) - Motor weakness 1.5% (3/205) - Post herpetic itching 1.0% (2/205) |
| New Delhi, Delhi (Gupta et al., 2011)^7^ (reference **17** of main text) | Institute of Medical Sciences | Retrospective chart review  Duration NR | - HZO† pts - Aged < 40 yrs - 18 pts - m:f 2.6:1 - Ages: 21–39 yrs - Mean age (SD): 30.3 (6.8) yrs - Chicken pox history: 27.8% (5/18) | - HIV: 44.4% (8/18) - TB: 11.1% (2/18) - Syphillis: 11.1% (2/18) - Vaginal Candida: 5.6% (1/18)   IC: 44.4% | NR | - Left side of the head & face lesions: 61.1% (11/18) - Only ophthalmic lesions: 73% (13/18) - Cervical and thoracic dermatomes: 27.8% (5/18, all HIV+) | NR | NR | - PHN: 38.9% (7/18) - Corneal involvement: 83.3% (15/18) - Upper lid cicatricial entropion: 16.7% (3/18) - Lower lid cicatricial ectropion: 11.1% (2/18) - Leukoma: 16.7% (3/18) - Nebular / Nebulomacular corneal opacity: 27.8% (5/18) - Leukomatous corneal opacity: 5.6% (1/18) - Dry eye: 5.6% (1/18) - Lid coloboma: 5.6% (1/18) - Focal loss of filia: 5.6% (1/18) - Secondary glaucoma: 27.8% (5/18) |
| Chennai, Tamil Nadu (Sundaram et al., 2012)^8^ (reference **40** of main text) | Dermatology Dpt of Tertiary Care hospital | Cross-sectional study  1 year | - HZ† pts - All ages - m:f 2.2:1 - Ages: 4–80 yrs | HIV: 32.3%  IC: 32.3% | NR | Dermatomal vesicular eruptions:^   - Thoracic: 55.0% - Cranial: 21.5% - Lumbar: 18.4% - Cervical: 4.6% - Sacral: 1.5% | NR | NR | NR |
| Adichunchanagiri, Karnataka (Maiya and Shenoy, 2013)^9^ (reference **32** of main text) | Ophthalmology Dpt of Institute of Medical Sciences | Prospective observational study, 1-year follow-up  1 year | - HZO† pts - All ages - 27 pts - m:f 0.8:1 - Ages: 11-90 yrs - Aged >50 yrs: 55.6% (15/27) - Aged >60 yrs: 18.5% (5/27) | - HIV: 11.1% (3/27) - DM: 7.4% (2/27) - Malaria: 3.7% (1/27) | Rash: 100% (27/27)  Eye watering: 33.3% (9/27)  Lid swelling: 18.5% (5/27)  Diminution of vision: 37.0% (10/27) | - Vesicular lesions on face 100% (27/27) - Right eye involvement: 55.6% (15/27) - Left eye involvement: 44.4% (12/27) | Neuralgia: 100% (27/27) | NR | 1. Overall:   PHN: 14.8%  Substantial visual loss: 33.3% (9/27)  Mild visual loss: 29.6% (8/27)   1. In HZO patients with ocular involvement (16/27):  - Anterior uveitis: 37.5% (6/16) - Punctate keratitis: 31.2% (5/16) - Secondary glaucoma: 18.8% (3/16) - Follicular conjunctivitis: 12.5% (2/16) - Keratouveitis: 6.2% (1/16) |
| Dharwad, Karnataka (Naveen et al., 2016)^10^ (reference **23** of main text) | Dermatology Dpt of College of Medical Sciences and Hospital | Prospective cross-sectional study  Jan 2012–Jan 2014 | - HZO† pts - All ages - 25 pts - m:f 2.1:1 - Ages: 18–75 yrs - Mean age (SD): 46.8 yrs - Aged> 50 yrs: 44.0% (11/25) - Aged >60 yrs: 28.0% (7/25) | - HIV [IC]: 16.0% (4/25) - DM: 16.0% (4/25) - Hypertension: 8% (2/25) - Hypotension: 4% (1/25) | Decreased or absent corneal sensation: 52.0% (13/25)  Dendritic ulcer: 12.0% (3/25)  Stromal keratitis: 12.0% (3/25)  Punctate keratopathy: 4% (1/25)  Anterior uveitis: 40% (10/25) | - Right side face vesicular lesions: 44.0% (11/25) - Left side face vesicular eruptions: 56.0% (14/25) - Disseminated HZ: 4% (1/25) | NR | NR | - Eyelid involvement: 76.0% (19/25) - Corneal involvement: 52.0% (13/25) - Anterior uveitis: 40.0% (10/25) - Impaired vision: 40.0% (10/25) |
| Hyderabad, Telangana (Katakam et al., 2016)^11^ (reference **18** of main text) | Dermatology Dpt of Medical College and Hospital | Prospective observational study, NR follow-up  Jan 2013–Dec 2014 | - HZ† pts - Aged < 18 yrs - 26 pts - m:f 0.9:1 - ages: 3–17 yrs - Chicken pox history: 53.8% (14/26) - Vaccinated (varicella): 30.8% (8/26) | HIV [IC]: 15.4% (4/26) | Prominent in 30.8% patients aged >12 yrs (8/26) | Dermatomal vesicular eruptions:^   - Thoracic: 53.8% (14/26) - Head: 15.4% (4/26) - Upper limbs: 15.4% (4/26) - Lower limbs: 15.4% (4/26) | - Mild pain in 2–12 yrs - Moderate pain in >12 yrs | NR | None |
| Kalaburagi, Karnataka (Malkud and Dyavannanavar, 2017)^12^ (reference **19** of main text) | Dermatology Dpt.ofTertiary Care Hospital. | Prospective cross-sectional study  Feb 2013–Feb 2016 | - HZ† children - Aged < 16 yrs - 30 pts - m:f 1.5:1 - Ages: 9 m–15 yrs - Chicken pox history: 43.3% (13/30) - Vaccinated (varicella): 16.7% (5/30) | - HIV 6.7% (2/30) - Steroid intake: 3.3% (1/30)   [IC: 10% (3/30)] | - Itching: 23.3% (7/30) - Burning sensation: 13.3% (4/30) | Dermatomal vesicular eruptions:^   - Thoracic: 56.7% (17/30) - Lumbar: 20.0% (6/30) - Cervical: 16.7% (5/30) - Cranial 3.3% (1/30) - Sacral 3.3% (1/30) | Pain in 13.3% patients (4/30) | NR | NR |
| Bemina, Jammu and Kashmir (Lanker et al., 2015)^13^ (reference **33** of main text) | Pediatrics & dermatology Dpts of Tertiary Care Hospital | Prospective cross-sectional observational study  April 2013–March 2014 | - HZ† pts - Aged ≤18 yrs - 19 pts - m:f 1.4:1 - Ages: 2–17 yrs - Median age: 10 yrs - Chicken pox history: 36.8% (7/19) - Vaccinated (varicella): 0% (0/19) | Steroid intake: 10.5%  [IC: 10.5% (2/19)] | NR | Dermatomal vesicular eruptions:^   - Thoracic: 55.0% - Lumbar: 35.0% - Sacral: 5.0%   HZO: 5.0% | NR | NR | Overall in 26.3%:(5/19)   - SBI: 15.8%,(3/19) - PHN: 10.5% (2/19) |
| Bangalore, Karnataka (Aggarwal and Radhakrishnan, 2016)^14^ (reference **15** of main text) | Dermatology Dpt of Tertiary Care Hospital | Prospective, cross-sectional study  Jun 2013–May 2014 | - HZ† pts - All ages - 84 pts - m:f 6.6:1 - Mean age; 30 yrs - Ages: 7–77 yrs - Aged >50 yrs: 17.9% (15/84) - Aged >60 yrs: 8.3% (7/84) | - HIV: 3.6% (3/84) - Bronchial asthma: 1.2%, (1/84) - Hodgkins lymphoma: 1.2% (1/84)   IC: 6.0% (5/84) | NR | Dermatomal vesicular eruptions^   - Thoracic: 65.5% (55/84) - Cervical: 11.9% (10/84) - Cranial: 10.7% (9/84) | Overall: 63.1% cases (53/84):   - 0–10 yrs: 0.0% - 11–20 yrs: 42% (6/84) - 21–30 yrs: 66.0% (22/84) - 31–40 yrs: 62.5% (10/84) - 41–50 yrs: 80.0% (4/84) - 51–60 yrs: 75.0% (6/84) - >60 yrs: 71.0% (5/84) | NR | Motor involvement: 1.2% (1/84) |
| Bangalore, Karnataka (Rachana et al., 2017)^15^ (reference **39** of main text) | Dermatology Dpt of Medical College | Prospective observational study  June 2013–Sep 2014 | - HZ† pts - Ages 18–75 yrs - 72 pts - m:f 0.9:1 - Mean age (SD): 58 (18) yrs - Aged> 50 yrs: 54.2% (39/72) - Aged >60 yrs: 27.8% (20/72) | - DM: 21% - Hypertension: 18.5% - HIV: 6.8% - Cancer: 13.5% - DM+Hypertension:.6% | Pain: 100% | Dermatomal vesicular eruptions:^   - Thoracic: 38.9% (28/72) - Lumbar: 30.6% (22/72) - Trigeminal: 22.2% (16/72) - Sacral: 2.8% (2/72) - Cervical: 5.6% (4/72) | - Pricking: 54.2% (39/72) - Shooting: 27.8% (20/72) - Burning: 18.3% (13/72) | NR | NR |
| Gulbarga, Karnataka (Malkud et al., 2016) ^16^ (reference **20** of main text) | Dermatology Dpt of District Public Hospital | Prospective cross-sectional observational study  June 2013–June 2015 | - HZ† pts - All ages - 240 pts - m:f 1.5:1 - Aged > 50 yrs: 31.7% (76/240) - Aged > 60 yrs: 16.7% (40/240) - Chicken pox history: 64.6% (155/240) | - Steroid intake: 11.7% (28/240) - Malignancies / chemotherapy: 6.7% (16/240) - DM: 5.8% (14/240) - HIV 4.2% (10/240) - Chronic renal failure 0.8% (2/240)   [IC:30.0% (72/240)] | - Fever, headache, and arthralgia: 10.0% (24/240) - Segmental neuralgia 91.7% (220/240) | Dermatomal vesicular eruptions:^   - Thoracic: 44.2% (106/240) - Cranial: 24.6% (59/240) - Cervical: 12.5% (30/240) - Lumbar: 9.2% (22/240) - Sacral: 6.2% (15/240) | Pain :   - Preceding vesicles: 60.4% (145/240) - Concurrent to vesicles: 35.4% (85/240) - 2–3 days after vesicles development: 4.2% (10/240) - Continuous burning pain: 4.2% (10/240) - Intermittent radicular pain: 6.3% (15/240) | NR | Overall 33.8% (81/240) had complications:   - SBI: 12.5% (30/240) - PHN: 10.4% (25/240) - Motor weakness: 0.8% (2/240) - Depigmentation: 2.1% (5/240) - Poste herpetic itching: 2.1% (5/240) - Scarring: 4.2% (10/240) - Severe ulceration: 1.7% (4/240) |
| Dibrugarh, Assam (Adhicari and Agarwal, 2017)^17^ (reference **28** of main text) | Dermatology Dpt of Medical College & Hospital | Prospective observational study, 4 months follow-up  July 2013–June 2014 | - HZ† pts - All ages - 113 pts - m:f 1.7:1 - Mean age (SD): 45.8 (NR) yrs - Ages: 12–80 yrs - Aged >50 yrs: 33.6% (38/113) - Aged >60 yrs: 18.6% (21/113) - Chicken pox history: 79.6% (90/113) | - DM: 9.7% (11/113) - Immunosuppressive drugs: 4.4% (5/113) - TB 2.7% (3/113) - HIV: 0.9% (1/113) | 61.9% (70/113) pts had prodromal symptoms  Burning sensation 22.1% (25/113)  Watering from eye 8.0% (9/113)  Itching 8.0% (9/113)  Paraesthesia 6.2% (7/113)  Fever 5.3% (6/113)Tingling 5.3% (6/113)  Headache 4.4% (5/113)  Presenting symptoms:   - Skin eruptions: 99.1% - Pain: 90.3% | Dermatomal vesicular eruptions:^   - Thoracic: 45.1% (51/113) - Cranial: 28.3% (32/113) - Lumbar: 15.0% (17/113) | Overall: 90.3% (102/113)) cases:   - Burning pain: 46.0% (52/113) - Stabbing pain: 24.8% (28/113) - Shooting pain 11.5% (13/113) | NR | Overall: 38.1 % (43/113) cases:   - PHN: 14.2% (16/113) - Pigmentation: 9.7% (11/113) - Scarring: 7.1% (8/113) - SBI: 3.5% (4/113) - Keloid: 1.8% (2/113) - Dissemination: 0.9% (1/113) |
| East India (Mondal et al., 2019)^18^ (reference **22** of main text) | Dermatology Dpt of two Tertiary Care Hospitals | Prospective cross-sectional observational study  1 year | - HZ† pts - All ages - 90 pts - m:f 1.9:1 - Mean age (SD): 29.6 (17.5) yrs - Ages: 8 m–75 yrs   Chicken pox history:   - Overall populations: 61.1% (55/90) | - High blood sugar 11.1% (10/90) - Steroids intake: 2.2% (2/90) - HIV: 0% | - Pain: 87.8% - Itching: 45.5% (41/90) - Fever: 44.4% (40/90) - Pain + burning sensation before lesions eruption: 33.3% (30/90) - Myalgia 27.8% (25/90) | Dermatomal vesicular eruptions:^   - Thoracic: 46.7% (42/90) - Abdomen: 31.1% (28/90) - Head and neck 14.4% (13/90) - Extremities: 7.8% (7/90) | Pain in 87.8% (79/90):   - Mild: 17.8% (16/90) - Moderate: 38.9% (35/90) - Severe: 31.1% (28/90)  \| Pain at presentation (% of cases) \| \| \| \| --- \| --- \| --- \| \|  \| Children (n=21) \| Adults (n=69) \| \| Mild \| 28.6% \| 14.5% \| \| Moderate \| 14.3% \| 46.4% \| \| Severe \| 23.8% \| 33.3% \| | NR | - SBI: 20% (18/90) - PHN: 14.5% adults (10/90) - Post inflammatory hyperpigmentation: (NR %) |
| Ludhiana, Punjab (Puri, 2016)^19^ (reference **26** of main text) | Dermatology Dpt of District Hospital | Prospective cross-sectional observational study  Duration NR | - HZ† pts - All ages - 50 pts - m:f 2.6:1 - Aged >60 yrs: 10% (5/50) | HIV [IC]: 8.0% (4/50) | - Fever 10.0% (5/50) - Paraesthesia 50.0% (25/50) - Itching 24.0% (12/50) | HZO 16.0% (/50)  Dermatomal vesicular eruptions:^   - Thoracic: 40.0% (20/50) - Trigeminal nerve area 24.0% (12/50) - Lumbar: 16.0% (8/50) - Cervical: 8.0% (4/50) - Sacral: 4.0% (2/50) | - Pain: 90% (45/50) | Pain persisting for three months or longer | - PHN: majority of adults >50 yrs - SBI: 10.0% (5/50) - Ulceration and necrosis: 10.0% (5/50) - Ramsay Hunt syndrome: 16.0% (8/50) |
| Srinagar, Jammu and Kashmir (Nusratnazirmakroo et al., 2017)^20^ (reference **30** of main text) | Oral Medicine & Radiology Dpt of Governmental College and Hospital | Prospective observational study, 4 months follow-up  Jan 2015–Feb 2016 | - HZ† pts - All ages - 56 pts - m:f: 1.8:1 - Aged >60 yrs: 23.2% | NR | NR | Vesicular lesions on face,  Buccal lesions | A few experienced severe pain | NR | - PHN: 33.9% (19/56) - Paraesthesia: 30.4% (17/56) - Itching 8.9% (5/56) - Ramsay Hunt syndrome: 7.1% (4/56) - SBI 3.6% (2/56) |
| Shimla, Himachal Pradesh (Gupta and Sareen, 2017)^21^ (reference **37** of main text) | Ophthalmology Dpt of District hospital | Prospective cross-sectional study  Jan 2015–Dec 2016 | - HZO† patients - All ages - 73 pts - m:f 1.9:1 - mean age (SD): 55.2 (10.5) - Ages: 34–85 yrs - Aged >50 yrs: 71.2% (52/73) - Aged > 60 yrs: 31.5% (23/73) | - DM: 19.2% (14/73) - HIV: 4.1% (3/73) - TB: 8.2% (6/73) - Malignancy: 1.4% (1/73) - Immunosuppressive drugs: 4.1% (3/73) - Others: 1.4% (1/73) | - Pain: 71.2% (52/73) - Watery eye: 58.9% (43/73) - Lid swelling: 56.2% (41/73) - Decreased vision: 35.6% (26/73) | Dermatomal vesicular eruptions on face (branches of ophthalmic nerve)   - Frontal: 100% (73/73) - Nasociliary: 39.7% (29/73) - Lacrimal: 35.6% (26/73) | Pain 71.2% (52/73)   - Unilateral - Lancinating - Burning | Pain persisting for three months or longer | - PHN: 24.6% (after 3 months) - Severe visual impairment: 2.7% - Secondary glaucoma: 8.2% |
| Burla, Odisha (Behera et al., 2019)^22^ (reference **35** of main text) | Ophthalmology Dpt of Tertiary Care Centre | Prospective observational study, 1 yr. follow-up  July 2016–Dec 2018 | - HZO† pts - All ages - 32 pts - m:f 1.7:1 - Mean age (SD): 57.3 (NR) - Ages 31–80 yrs - Aged >50 yrs: 81.3% (26/32) - Aged >60 yrs: 62.5% (20/32) | - DM: 56.3% (18/32) - HIV: 18.8% (6/32) - Anemia; 1% (4/32) - Hepatitis B: 9.4% (3/32) - Chemotherapy: 3.1% (1/32)   IC:21.9% (7/32) | Periorbital rash: 100% (32/32)  Punctate keratitis: 93.8% (30/32)  Lid edema: 87.5% (28/32)  Conjunctivitis: 78.1% (25/32)  Conjunctival edema: 56.3% (18/32)  Uveitis: 56.3% (18/32) | Face lesions:   - Right side: 75% (24/32) - Left side: 25% (8/32) | …… | Pain present at the end of the 2-month follow up | - PHN 15.6% (5/32) - Variably impaired visual acuity 100% (32/32) - Secondary glaucoma: 28.1% - Progressive outer retinal necrosis 6.2% |
| NR (Mitra et al., 2018)^23^ (reference **21** of main text) | Pediatrics & Dermatology Dpt of Tertiary Care Hospital | Prospective cohort study, 1 month follow-up  2 yrs duration | - HZ† children - Aged < 12 yrs - 39 pts - m:f 0.8:1 - Ages: 3–11 yrs - Median age: 4.5 yrs - Chicken pox history: 28.2% (11/39) - Vaccinated (varicella): 30.8% (12/39) | - HIV: 5.1% (2/39) - Leukaemia: 2.6% (1/39)   IC: 7.7% | None | - Thoracic: 51.3% (20/39) - Upper limbs: 23.1% (9/39) - Head and neck: 15.4% (6/39) - Lower limbs: 10.3% (4/39) | Mild burning sensation & pain | NR | None |
| Gangtok, Sikkim (Sharma and Sharma, 2019)^24^ (reference **29** of main text) | Dermatology Dpt of Central Referral Hospital | Prospective cross-sectional observational study  Jan 2018–Dec 2018 | - HZ† pts - All ages - 109 pts - m:f 1.5:1 - Aged >50 yrs: 35.8% (39/109) - Aged >60 yrs: 22.0% (24/109) | - Immunosuppressives: 11.0% (12/109) - DM: 10.1% (11/109) - Hypertension: 9.2% (10/109) - Hepatic and GI disorders: 6.4% (7/109) - Arthritis: 4.6% (5/109) - Asthma and COPD: 3.7% (4/109) - Carcinoma: 2.8% (3/109) - Hypothyroidism 2.8% (3/109) - Tinea infection: 1.8% (2/109) - Hyperthyroidism: 0.9% (1/109) | NR | Dermatomal vesicular eruptions^   - Multidermatomal 8.25% (9/109) - Thoracic 40.4% (44/109) - Cervical: 23.8% (26/109) - Cranial: 22.9% (25/109) - Sacral: 7.3% (8/109) - Lumbar: 5.5% (6/109)   Lesions:  Right side: 54.1% (59/109)  Left side: 43.1% (47/109) | NR | NR | NR |
| Ongole, Andhra Pradesh (Naik, 2019)^25^ (reference **34** of main text) | Dermatology Dpt of Institute of Medical Sciences | Prospective cross-sectional observational study  Oct 2018–Aug 2019 | - HZ† patients - All ages - 116 pts - m:f 0.6:1 - Aged > 50 yrs: 47.4% (55/116) - Aged >60 yrs: 17.2% (20/116) | - DM:11.2% (13/116) - HIV: 4.3% (5/116) - Steroid intake: 1.7% (2/116) - HIV with TB coinfection: 0.9% (1/116) - COPD: 0.9% (1/116)   IC: 19.0% (22/116) | - Burning sensation: 68.9% (80/116) - Fever: 12.9% (15/116) - Itching: 10.3% (12/116) - Paraesthesia 7.7% (9/116) | Dermatomal vesicular eruptions:^   - Thoracic: 53.4% (62/116) - Cervical: 19.0% (22/116) - Trigeminal (facial): 17.2% (20/116) - Lumbar: 6.0% (7/116) - Sacral: 4.3% (5/116) | - Pain: 74.1% (86/116) - Ear pain: 3.4% cases (4/116) | Pain present for 90 days or more after rash onset | - PHN: 28.4% (33/116) - Sensory loss: 24.1% (28/116) - Ocular complications: 19.8% (23/116) - SBI: 19.0% (22/116) - Scarring: 14.6% (17/116) - Hearing loss: 12.1% (14/116) - Pneumonia: 6.9% (8/116) |
| Tirupati, Andhra Pradesh (Usha et al., 2015)^26^ (reference **36** of main text) | Dermatology Dpt of General Public Hospital | Prospective cross-sectional observational study  Duration NR | - HZ† pts - All ages - 100 pts - m:f 2.2:1 - Aged >50 yrs: 15.0% (15/100) - Aged >60 yrs: 5.0% (5/100) | - HIV [IC]: 32.0% (32/100) - DM: 3.0% (3/00) | Overall in 34.0% (34/100) | Dermatomal vesicular eruptions:^   - Thoracic: 51.0% (51/100) - Cervical: 21.0% (21/100) - Lumbosacral: 16.0% (16/100) - Cranial: 12.0% (12/100) - Multidermatomal: 42.0% (42/100) | …….. | NR | - SBI:21.0% (21/100) - PHN: 15.0% (15/100) - Pigmentary changes: 15.0% (15/100) - Scarring with milia: 2.0% (2/100) - Disseminated disease: 1.0% (1/100) - Keloid: 1.0% (1/100) - Brachial amyotrophy: 1.0% (1/100) |
| Bangalore, Karnataka (Shanthaveerappa and Parappallil, 2019)^27^ (reference **38** of main text) | Ophthalmology Dpt of University Medical Hospital | Prospective observational study, 1-month follow-up  1 year | - HZO† pts - All ages - 20 pts - m:f 1.9:1 - Mean age (SD): 49.5 (NR) - Ages: 14–75 yrs - Aged >50 yrs: 60.0% (12/20) - Aged >60 yrs: 25.0% (5/20) | DM: 45.0% (9/20)  HIV: 0% (0/20) | - Watering eyes: 50.0% (10/20) - Lid swelling: 40.0% (8/20) - Diminution of vision: 25.0% (5/20) | Dermatomal vesicular eruptions on face:   - Ocular involvement: 80.0% (16/20) - Skin rash: 100% (20/20) | Acute neuralgia: 100% (20/20) | Pain noted at 1-month follow up | - PHN 40.0% (8/20) - Lid scarring 30.0% (6/20) - Follicular conjunctivitis 15.0% (3/20) - Punctate keratitis 15.0% (3/20) - Disciform keratitis 10.0% (2/20) - Dendritic ulcer 5.0% (1/20) - Secondary glaucoma 15.0% (3/20) - Persistent synechiae 5.0% (1/20) - Ptosis 5.0% (1/20) |

COPD, chronic obstructive pulmonary disease; DM, diabetes mellitus; Dpt, department; GI, gastrointestinal; HIV, human immunodeficiency virus;; HZ, herpes zoster; HZO, herpes zoster ophthalmicus;; m, months; m:f, male:female ratio; n, number of subjects; NR, not reported; PHN, Post Herpetic Neuralgia; pts, patients; SBI, secondary bacterial infection; TB, tuberculosis; SD, standard deviation; yrs, years

*best estimation based on any relevant descriptions provided within each respective publication

† HZ and HZO cases were clinically diagnosed in most studies; few had Tzanck smear and other tests done

**defined as herpetic pain persistent one month following rash resolution; ††defined as pain persistent >3 months from HZO onset

^ Commonest dermatomes one or more- thoracic, cranial, cervical, lumbar, face

1. Nithyanandam S, Stephen J, Joseph M, Dabir S. Factors affecting visual outcome in herpes zoster ophthalmicus: a prospective study. *Clinical & experimental ophthalmology.* 2010;38(9):845-850.

2. Naveen KN, Tophakane RS, Hanumanthayya K, Pv B, Pai VV. A study of HIV seropositivity with various clinical manifestation of herpes zoster among patients from Karnataka, India. *Dermatology online journal.* 2011;17(12):3.

3. Singh GK, Singh Deora M, Grewal R, Kushwaha A, Minhas S. Is High Altitude a Risk Factor in Development of Herpes Zoster? *High altitude medicine & biology.* 2018;19(3):244-248.

4. Babu K, Mahendradas P, Sudheer B, et al. Clinical Profile of Herpes Zoster Ophthalmicus in a South Indian Patient Population. *Ocular immunology and inflammation.* 2018;26(2):178-183.

5. Vora RV, Singhal RR, Anjaneyan G, Patel TM. Clinicoepidemiological study of herpes zoster at rural based tertiary center of Gujarat. *IP Indian Journal of Clinical and Experimental Dermatology.* 2018;4(1):40-43.

6. Abdul Latheef E, Pavithran K. Herpes zoster: A clinical study in 205 patients. *Indian Journal of Dermatology.* 2011;56(5):529-532.

7. Gupta N, Sachdev R, Sinha R, Titiyal JS, Tandon R. Herpes zoster ophthalmicus: disease spectrum in young adults. *Middle East African journal of ophthalmology.* 2011;18(2):178-182.

8. Sundaram M, Adikrishnan S, Krishnakanth M, et al. Hospital based cross sectional study of herpes zoster with reference to HIV seropositivity. *BMC Infect Dis.* 2012;12:P58.

9. Maiya AS, Shenoy S. A Clinical Study of Herpes Zoster Ophthalmicus. *IOSR Journal of Dental and Medical Sciences.* 2013;12(6):9-13.

10. Naveen KN, Pradeep AV, Athanker SB. A study of clinical profile and ophthalmological manifestations of herpes zoster ophthalmicus with HIV seropositivity in Northern Karnataka. *Journal of Pakistan Association of Dermatologists.* 2016;26(1):21-25.

11. Katakam BK, Kiran G, Kumar U. A Prospective Study of Herpes Zoster in Children. *Indian J Dermatol.* 2016;61(5):534-539.

12. Malkud S, Dyavannanavar V. Childhood herpes zoster: A study from tertiary center. *Journal of Pakistan Association of Dermatologists.* 2017;27(2):120-123.

13. Lanker AM, Jeelani S, Jeelani N. Herpes zoster in the pediatric age group: study from a tertiary care hospital. *International Journal of Contemporary Pediatrics.* 2015;2(4):321-324.

14. Aggarwal SK, Radhakrishnan S. A clinico-epidemiological study of herpes zoster. *Medical journal, Armed Forces India.* 2016;72(2):175-177.

15. Rachana R, Shivaswamy KN, Anuradha HV. A study on clinical characteristics of herpes zoster in a tertiary care center. *International Journal of Research in Dermatology.* 2017;3(1):79-82.

16. Malkud S, Dyavannanavar V, Purnachandra, Murthy KS. Clinical and morphological characteristics of herpes zoster - A study from tertiary care centre. *Journal of Pakistan Association of Dermatologists.* 2016;26(3):219-222.

17. Adhicari D, Agarwal D. A Hospital-Based Clinical Study of Herpes Zoster- A report of 113 cases. *IOSR Journal of Dental and Medical Sciences.* 2017;16:33-38.

18. Mondal A, Kumar P, Swathi G, Dasarathan S. A comparative study of herpes zoster: Adult versus paediatric patients. *Journal of Pakistan Association of Dermatologists.* 2019;29(3):278-285.

19. Puri N. A study on clinical presentation of herpes zoster in a district hospital in North India. *Journal of Pakistan Association of Dermatologists.* 2016;26(2):134-137.

20. Nusratnazirmakroo, Chalkoo AH, Peerzada G. A study based on clinical presentation and complications in Herpes Zoster patients: An analytical study. *Indian J Dent Adv.* 2017;9(1):24-28.

21. Gupta M, Sareen A. A Prospective Study to Observe Ocular Outcome and Clinicoepidemiological Pattern in Herpes Zoster Ophthalmicusat District Hospital in Hilly Area in India. *Scholars Journal of Applied Medical Sciences.* 2017;5(7B):2607-2614.

22. Behera S, Swain LM, Dora J, Pandey S. Herpes zoster ophthalmicus: clinical profile and management, a prospective study at a tertiary health care centre, Western Odisha. *International Journal of Scientific Research.* 2019;8(2):61-63.

23. Mitra B, Chopra A, Talukdar K, Saraswat N, Mitra D, Das J. A Clinico-epidemiological Study of Childhood Herpes Zoster. *Indian Dermatol Online J.* 2018;9(6):383-388.

24. Sharma R, Sharma R. Clinical study of herpes zoster in 109 patients in central referral hospital, Gangtok. *International Journal of Research in Dermatology.* 2019;5:849-852.

25. Naik B. Herpes zoster, shingles - A clinicoepidemiological study and its complications among immunocompetent and immunocompromised patients. *International Journal of Scientific Research.* 2019;8(10):69-71.

26. Usha G, Srinivasulu P, Bharathi G. Clinico Epidemiological Study of Herpes Zoster in HIV Era in a Tertiary Care Hospital in South India. *IOSR Journal of Dental and Medical Sciences.* 2015;14(2):32-35.

27. Shanthaveerappa P, Parappallil RJ. The Clinical Profile and Ocular Manifestations of Herpes Zoster Ophthalmicus - A Hospital Based Study. *International Journal of Ophthalmology & Visual Science.* 2019;4(1):19-23.

## Supplementary Figure 1. Flowchart of the literature search and selection of relevant records


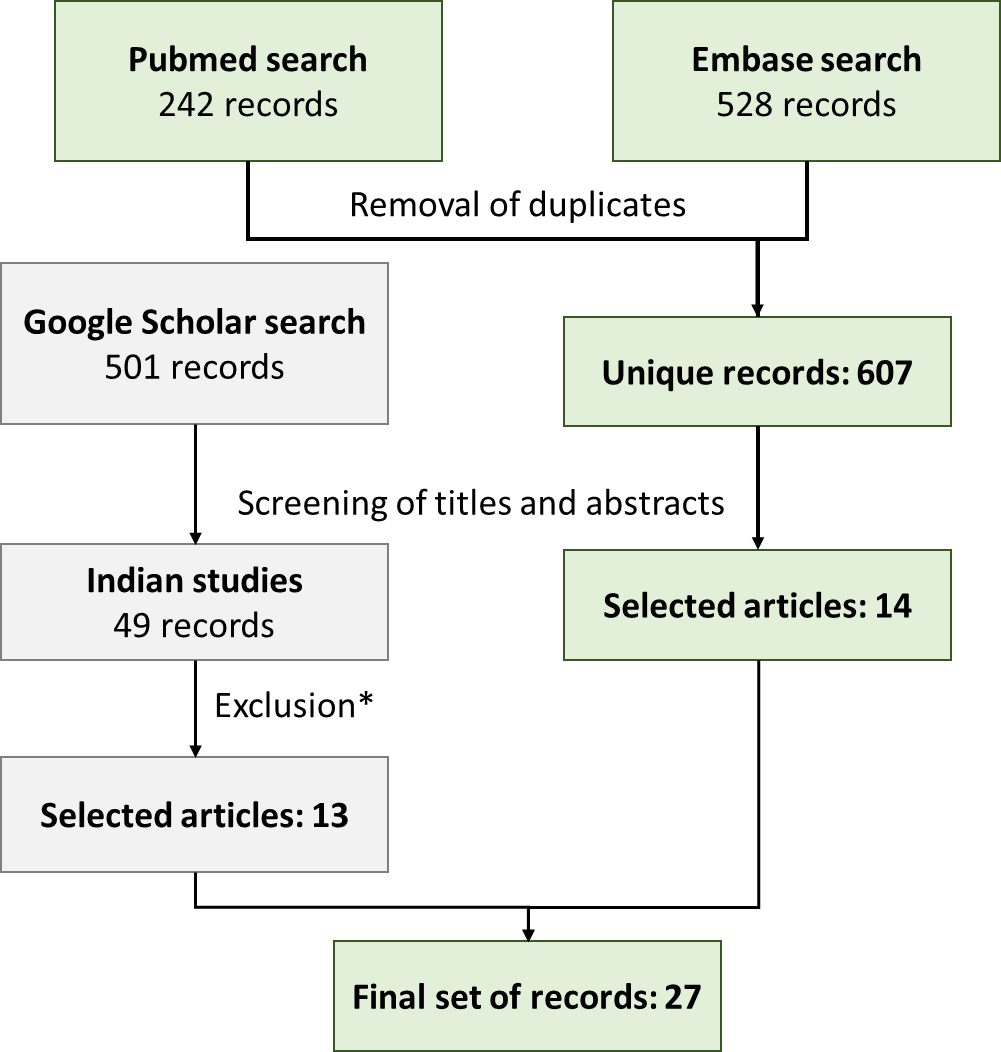


*Reasons for exclusion: did not meet inclusion criteria (n=19); not peer-reviewed (n=1); duplicates from Embase/Pubmed (n=11); inconsistencies in presented data (n=1); predatory journals/publishers (n=4).
