# Supplementary material for: Herpes zoster in outpatient departments of healthcare centers in India: a review of literature
Source: Hum Vaccin Immunother. 2021 Sep 14;17(11):4155–62. doi: 10.1080/21645515.2021.1968737 (PMC8828134; doi:10.1080/21645515.2021.1968737)
Supplement: Supplemental Material [file KHVI_A_1968737_SM7848.pdf]

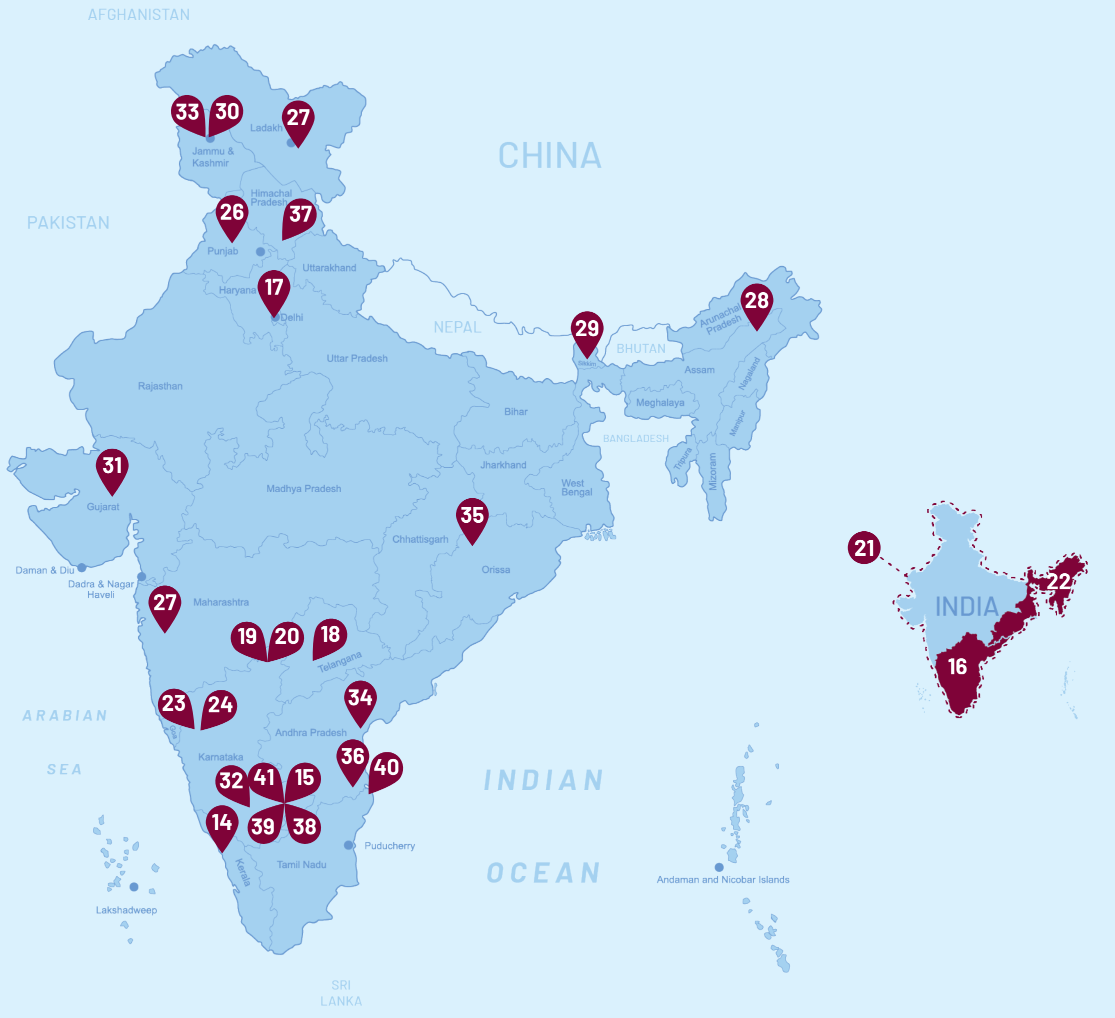

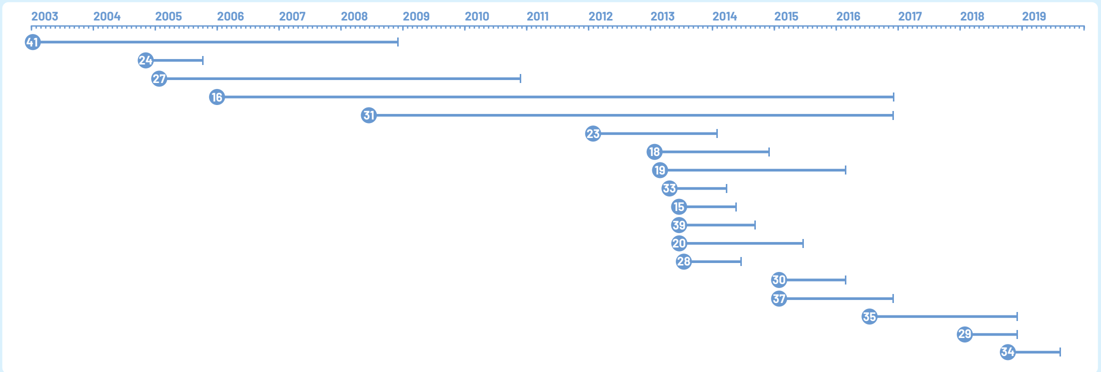

REF	City	Period of study	Number of cases (frequency)	Age range (mean-median)	Male:Female ratio
14	Calicut	2 years	205	3-85 years	1.3:1
15	Bangalore	June 2013 – May 2014	84	7-77 years (mean: 30)	6.6:1
16	South India	2006 – 2016	249	6-82 years (mean: 46.8)	1.1:1
17	New Dehli	NR	18	21-39 years (mean: 30.3)	2.6:1
18	Hyderabad	Jan. 2013 – Dec. 2014	26	3-17 years	0.9:1
19	Kalaburagi	Feb. 2013 – Feb. 2016	30	0.75 – 15 years	1.5:1
20	Gulbarga	June 2013 – June 2015	240	All ages	1.5:1
21	NR	2 years	39 (0.84)	3-11 years (median: 4.5)	0.8:1
22	East India	1 year	90 (0.28)	All ages (mean: 29.6)	1.9:1
23	Dharwad	Jan. 2012 – Jan. 2014	25	18-75 years (mean: 46.8)	2.1:1
24	Hubli	Nov. 2004 - Oct. 2005	90 (0.38)	4-72 years (mean: 38.8)	1.6:1
26	Ludhiana	NR	50	All ages	2.6:1
27	Leh and Pune	Jan. 2005 - Dec. 2010	239 (2.36 – 0.23)	20-60 years	Men only
28	Dibrugarh	July 2013 – June 2014	113 (0.51)	12-80 years (mean: 45.8)	1.7:1
29	Gangtok	Jan. 2018 - Dec. 2018	109	All ages	1.5:1
30	Srinagar	Jan. 2015 - Feb. 2016	56	All ages	1.8:1
31	NR	June 2008 - Dec. 2016	938 (0.38)	2-87 years (mean: 45.9)	1.4:1
32	Adichunchanagiri	1 year	27	11-90 years	0.8:1
33	Bemina	April 2013 - March 2014	19	2-17 years (median: 10)	1.4:1
34	Ongole	Oct. 2018 - Aug. 2019	116	All ages	0.6:1
35	Burla	July 2016 - Dec. 2018	32	31-80 years (mean: 57.3)	1.7:1
36	Tirupati	NR	100	All ages	2.2:1
37	Shimla	Jan. 2015 - Dec. 2016	73	All ages (mean: 55.2)	1.9:1
38	Bangalore	1 year	20	14-75 years (mean: 49.5)	1.9:1
39	Bangalore	June 2013 - Sep. 2014	72	18-75 years (mean: 58)	0.9:1
40	Chennai	1 year	NR (0.46)	4-80 years	2.2:1
41	Bangalore	Jan. 2003 - Dec. 2008	64	6-75 years (mean: 45.1)	1.6:1

REF: Reference   NR: Not reported
